# Comparison of *Mycoplasma hyopneumoniae* and porcine circovirus 2 commercial vaccines efficacy when applied separate or combined under experimental conditions

**DOI:** 10.1186/s40813-020-00148-0

**Published:** 2020-05-04

**Authors:** M. Sibila, G. Guevara, R. Cuadrado, P. Pleguezuelos, D. Pérez, A. Pérez de Rozas, E. Huerta, A. Llorens, O. Valero, M. Pérez, C. López, R. Krejci, J. Segalés

**Affiliations:** 1 IRTA, Centre de Recerca en Sanitat Animal (CReSA, IRTA- UAB), Campus de la Universitat Autònoma de Barcelona, Bellaterra, 08193 Barcelona, Spain; 2 OIE Collaborating Centre for the Research and Control of Emerging and Re-emerging Swine Diseases in Europe (IRTA-CReSA), Bellaterra, Barcelona, Spain; 3 Servei d’Estadística Aplicada, Universitat Autònoma de Barcelona, Campus de la Universitat Autònoma de Barcelona, Bellaterra, 08193 Barcelona, Spain; 4 Departament de Sanitat i Anatomia Animals, Universitat Autònoma de Barcelona, Bellaterra, 08193 Barcelona, Spain; 5 Ceva, La Ballastiere-BP, 126, 33501 Libourne Cedex, France; 6 UAB, Centre de Recerca en Sanitat Animal (CReSA, IRTA- UAB), Campus de la Universitat Autònoma de Barcelona, Bellaterra, 08193 Barcelona, Spain

**Keywords:** Porcine circovirus 2, *Mycoplasma hyopneumoniae*, Vaccine, Ready-to-use, Ready-to-mix

## Abstract

**Background:**

*Mycoplasma hyopneumoniae* (Mhyo) and *Porcine circovirus 2* (PCV-2) are two of the most significant infectious agents causing economic losses in the weaning to slaughter period. Due to their similar vaccination age, the objective of this study was to assess the efficacy of two already existing Mhyo (Hyogen®) and PCV-2 (Circovac®) vaccines when administered separately or combined (RTM) by means of Mhyo or PCV-2 experimental challenges.

**Results:**

Seven groups of animals were included in the study, being three of them challenged with PCV-2, three with Mhyo and one composed of non-challenged, non-vaccinated pigs. Within each experimental challenge, non-vaccinated (NV) groups were compared with double vaccinated groups using the commercial products separated (VS) or combined (VC). Both vaccinated groups showed significant differences for most parameters measured regarding PCV-2 (serology, percentage of infected animals and viral load in tissues) and Mhyo (serology and gross lesions) when compared to NV groups. VS and VC offered similar results, being only significantly different the PCV-2 antibody values at different time points (higher in the VS group) of the study, although not at the termination day (21 days post-PCV-2 inoculation).

**Conclusion:**

The present study expands the knowledge on the possibility of using two separate Mhyo and PCV-2 commercial vaccines as a RTM product, which offered equivalent virological, immunological and pathological outcomes as compared to these vaccines when used by separate.

## Background

Modern pig production sustainability, profitability and efficiency mainly depend on the zootechnical performance of farms, which in turn is affected by animal health and welfare [1]. Impairment of the health status, and in consequence of the animal welfare, is usually multifactorial, with both infectious and non-infectious factors able to exert detrimental effects.

Among infectious diseases, those causing respiratory and systemic problems account for an important percentage of losses in pig farms [2]. Economic losses are mainly attributed to mortality, growth retardation, medication associated costs and slaughter penalizations due to uneven size of animals. Although the variety of infectious agents linked to respiratory and systemic disorders is large, two of the most significant ones are *Mycoplasma hyopneumoniae* (Mhyo) and *Porcine circovirus 2* (PCV-2).

Besides, Mhyo is the etiological agent of enzootic pneumonia and PCV-2 the essential infectious cause for a group of diseases named porcine circovirus diseases (PCVD) [3]. Indeed, both pathogens can be associated with the so-called porcine respiratory disease complex (PRDC) [4]. PRDC is clinically characterized by coughing, dyspnea, poor growth and increased mortality [5]. Despite many other pathogens can also participate in PRDC [6], prevention and control of PCV-2 and Mhyo infections may represent corner-stones to approach this multifactorial disorder. This scenario is further emphasized by the fact that concomitant infections with PCV-2 and Mhyo are frequently found under field conditions [4] and a synergistic effect of both infections has been demonstrated in certain experimental models [7, 8].

The most common practice to prevent Mhyo infections is vaccination [9]. In fact, there is a high number of vaccine products marketed worldwide [9, 10], which are mainly applied from the first week of life onwards. Infection due to PCV-2 is almost uniquely prevented and controlled by means of vaccination, being applied mostly around weaning [11]. Although other interventions (biosecurity, diet, stocking density, genetics and management) may partially help in controlling PCVDs, PCV-2 vaccines offer the best efficacy by far [11]. Taking into account that the infection dynamics of both pathogens have some parallelisms (the peak of infection usually occurs during the postweaning period, although not necessarily concomitant), and that vaccine application is usually in the piglet, the concept of combined vaccination has been explored in the last 10 years. Such combined applications imply less handling labour and, therefore, saving in management associated costs. The first approach consisted of combining the two already existing commercial vaccines from the same manufacturer in a ready-to-mix (RTM) strategy [12], but lately ready-to-use (RTU) products have been developed and reached the market [13, 14]. Therefore, the aim of the present study was to assess the efficacy of two already existing products in the market, Mhyo (Hyogen®) and PCV-2 (Circovac®) vaccines, when administered separately or combined (RTM) by means of Mhyo or PCV-2 experimental challenges.

## Results

### Clinical signs and gross lesions

Four animals died before the end of the study. One animal from the VS (separate vaccination)-C (challenged) Mhyo group died during blood sampling on SD (study day) 0. Another one from the VC (combined vaccination)-C Mhyo group was euthanized on SD16 due to welfare reasons. This latter animal lost body condition and suffered from lameness of the left and right posterior limbs (tarsus). At necropsy, this pig showed absence of pulmonary collapse and mild increase of tarsal articular fluid. A swab from this joint was obtained and analysed by bacterial isolation without success*.* One animal from the VC-C PCV-2 group was found dead on SD15. At necropsy, this pig had pleuritis and fibrin in the thoracic cavity and yellowish fluid in the left anterior limb joints (carpus and elbow). *M. hyorhinis* was detected and cultured from swabs collected from joints. And finally, one animal from the NV (non-vaccinated)-C PCV-2 group was found dead (sudden death without clinical signs) on SD16. At necropsy, this pig had blood-stained liquid and fibrin in the abdominal and thoracic cavities. *Streptococcus suis* was isolated in pure culture from a peritoneal swab. All these animals were removed from the study.

### Body weight and ADWG

Mean body weight (±SD) and Average daily weight gain (ADWG) (±SD) per treatment and time-point are detailed in Table 1. No statistically significant differences neither in body weight nor ADWG through the study were observed between treatments in each challenge experiment.

**Table 1:** Mean body weight (±SD) and mean ADWG (±SD) per treatment group

**PCV-2 experiment treatment groups**	Body Weight (Kg)			ADWG (Kg)		
	SD-5	SD62	SD83	ADWG SD5-SD62	ADWG SD62-SD83	ADWG SD5-SD83
**NV-NC**	3.80 ± 0.85	41.95 ± 8.75	63.43 ± 9.25	0.57 ± 0.12	1.02 ± 0.10	0.68 ± 0.10
**NV-C PCV-2**	3.81 ± 0.68	38.80 ± 6.31	60.61 ± 7.56	0.52 ± 0.09	1.04 ± 0.13	0.65 ± 0.08
**VS-C PCV-2**	3.69 ± 0,49	37.25 ± 6.16	56.96 ± 8,15	0.50 ± 0.09	0.94 ± 0.19	0.61 ± 0.09
**VC-C PCV-2**	3.65 ± 0.52	37.31 ± 4.27	57.61 ± 5.52	0.50 ± 0.06	0.97 ± 0.11	0.61 ± 0.06
**Mhyo experiment treatment groups**	Body Weight (Kg)			ADWG (Kg)		
	SD-5	SD62	SD91	ADWG SD5-SD62	ADWG SD62-SD91	ADWG SD5-SD91
**NV-NC**	3.80 ± 0.85	41.95 ± 8.75	70,22 ± 4.66	0.57 ± 0.12	1.05 ± 0.11	0.69 ± 0.05
**NV-C Mhyo**	3.77 ± 0.65	39.51 ± 4.96	66.09 ± 10.67	0.53 ± 0.07	0.92 ± 0.25	0.65 ± 0.11
**VS-C Mhyo**	3.77 ± 0.63	36.97 ± 4.30	64.48 ± 5.93	0.50 ± 0.06	0.95 ± 0.11	0.63 ± 0.06
**VC-C Mhyo**	3.81 ± 0.69	41.06 ± 4.86	70.33 ± 5.85	0.55 ± 0.06	1.01 ± 0.07	0.69 ± 0.06

### PCV-2 results

#### Detection of antibodies against PCV-2

**Fig. 1:**
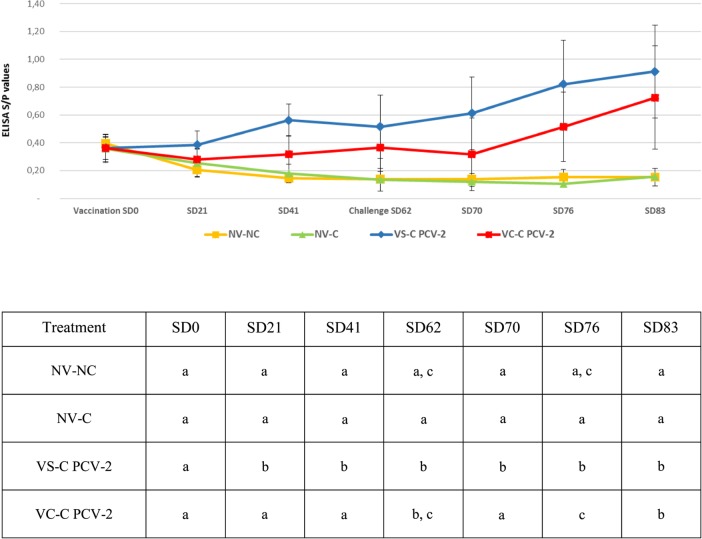
Mean (±SD) PCV-2 S/P ELISA values per treatment group and sampling point. Statistical differences are represented in the table below. Different letters in superscript within a column means *p* < 0.05

Mean Sample/Positive (S/*P)* ELISA values (±SD) per treatment group and sampling point are represented in Fig. 1. No statistically significant differences between treatment groups in mean S/P enzyme-linked immunosorbent assays (ELISA) values at the time of treatment distribution (data not shown) and vaccination were found. From SD21 until the end of the study, pigs from the VS-C PCV-2 group showed significantly higher S/*P* values than NV-NC and NV-C groups. VS-C PCV-2 group showed higher S/P values than VC-C PCV-2 group, being these differences statistically significant at SD21, SD41 SD70 and SD76.

#### Detection and quantification of PCV-2 DNA

**Fig. 2:**
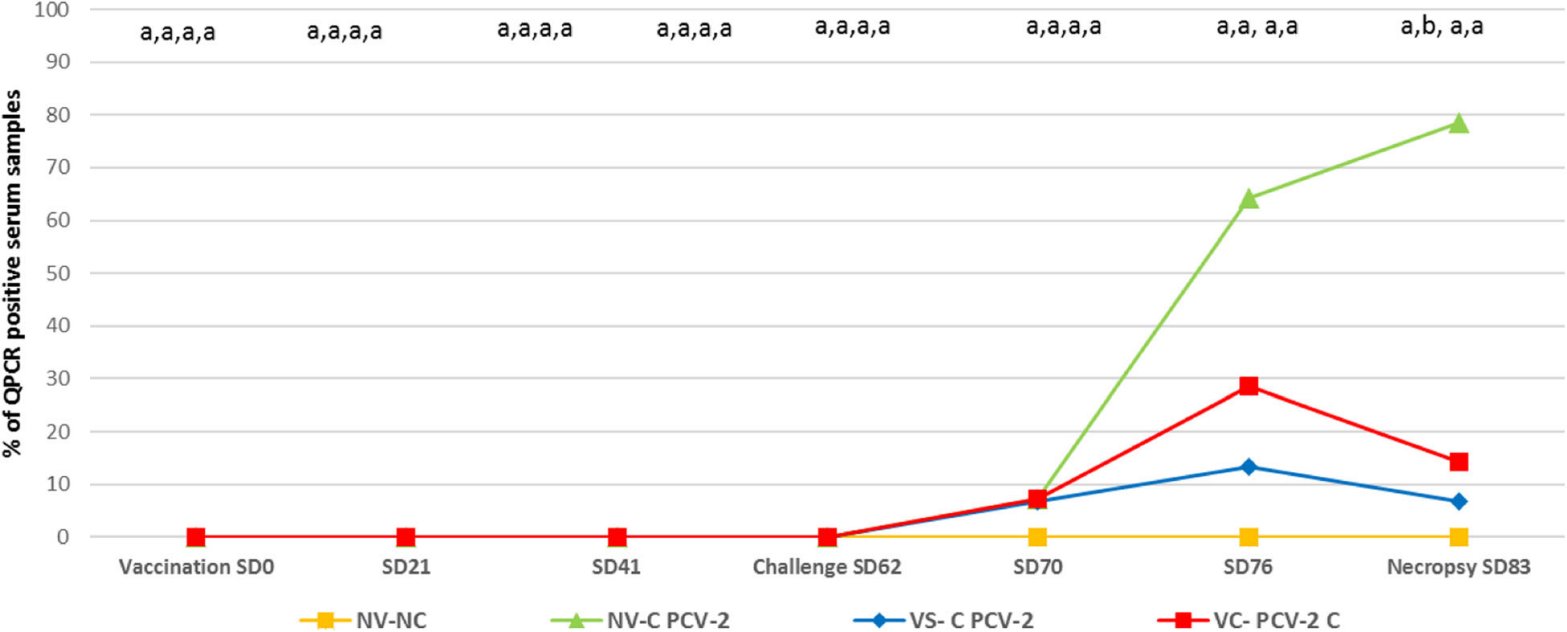
PCV-2 QPCR results expressed in percentage of positive serum samples per treatment group and sampling point. Different letters in superscript means *p* < 0.05

All animals included in the NV-NC group remained quantitative real-time polymerase chain reaction (QPCR) negative throughout the study. PCV-2 was firstly detected at 7 days post-inoculation (dpi) (SD70), where percentages of detection in all challenged groups were below 10% (Fig. 2). A higher percentage of QPCR positive serum samples was observed in the NV-C PCV-2 group at SD76 (9/14 [64,3%]) and SD83 (11/14, [78,6%]) compared to VC-C PCV-2 (4/14 [28,6%] and 2/14 [14,3%], respectively) and VS-C PCV-2 (2/15 [13,4%] and 1/14 [6,7%], respectively) groups. These differences, however, were only statistically significant on SD83. No statistically significant differences were found on percentage of PCV-2 QPCR pigs between vaccinated groups at any sampling point.

**Fig. 3:**
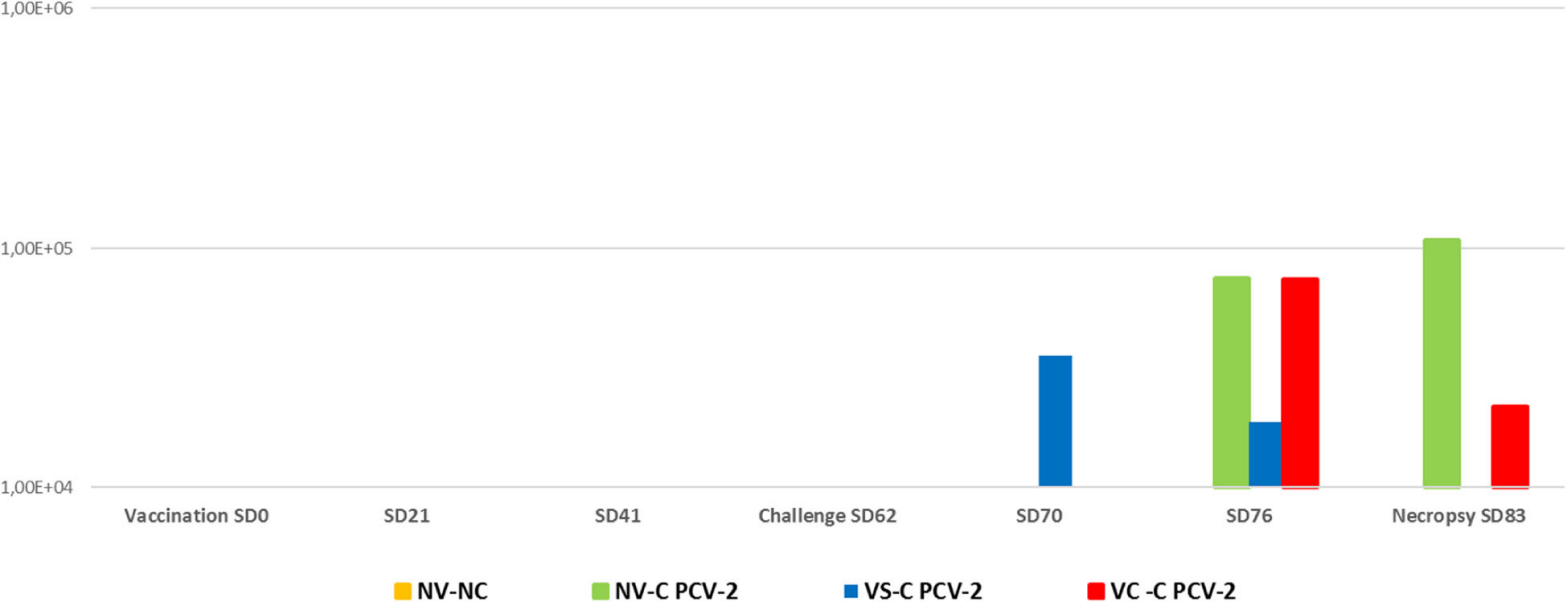
Mean PCV-2 copies/mL in PCV-2 QPCR positive samples per treatment group and sampling point

PCV-2 viral load within QPCR positive samples ranged from 1 × 10^4^ to 5.26 × 10^5^ copies/mL (Fig. 3). No statistically significant differences on the mean PCV-2 copies/mL of PCV-2 QPCR positive samples was found between treatment groups at any sampling point.

Animals from NV-C group had a numerically higher area under the curve (AUC) (5.1) than animals from VC-C PCV-2 (5.0) and VS-C PCV-2 (4.2) groups. These values became statistically significant when all the animals (positive and negative to QPCR) from each group were included (data not shown).

#### PCV-2 macroscopic lesion assessment, histopathological analyses and PCV-2 immunohistochemistry (IHC) results

No relevant gross findings were observed during the necropsies. In addition, all lymphoid tissue samples analysed scored 0 for lymphocyte depletion (LD) as well as for histiocytic infiltration (HI). On the other hand, all lymphoid tissue samples from animals included in groups NV-NC and VS-C PCV-2 scored 0 by IHC (Table 2). The NV-C PCV-2 group had a significantly higher number of animals with IHC scoring ≥1 in Tracheobronchial lymph node (TBLN) (8/14, 57.1%) than the VS-C PCV-2 group (0/15, 0%). Additionally, the number of animals with at least one tissue with score ≥ 1 was significantly higher in the NV-C PCV-2 group (9/14, 64.2%) when compared to VS-C PCV-2 (0/15) and VC-C PCV-2 (2/14, 14.3%) groups (Table 2). No significant differences on the number of lymphoid tissues scoring ≥1 between both vaccinated groups were detected.

**Table 2:** Number of animals with a IHC score ≥ 1 per lymphoid tissue and number of animals with at least one tissue scored ≥1 per treatment group

Treatment	Number of animals with a IHC score ≥ 1 per lymphoid tissue				Number of animals with at least one tissue scored ≥1 per treatment group
	TO	TBLN	MSLN	ISLN	
**NV-NC**	0^a^	0^a^	0 ^a^	0 ^a^	0^a^ (0%)
**NV-C PCV-2**	5^a^ (35.7%)	8^b^ (57.1%)	2^a^ (14.3%)	4^a^ (28.6%)	9^b^ (64.2%)
**VS-C PCV-2**	0^a^	0 ^a^	0^a^	0^a^	0^a^ (0%)
**VC-C PCV-2**	1^a^ (7.1%)	2^a, b^ (14.3%)	1^a^ (7.1%)	1^a^ (7.1%)	2^a^ (14.2%)

Different letters in superscript within a column means *p* < 0.05

*TO* Tonsil, *TBLN* Tracheobronchial lymph node, *MSLN* Mesenteric lymph node, *ISLN* Superficial inguinal lymph node, *LD* Lymphocyte depletion, *HI* Histiocytic infiltration, *IHC* Immunohistochemistry

### Mhyo results

#### Detection of antibodies against Mhyo 

**Fig. 4:**
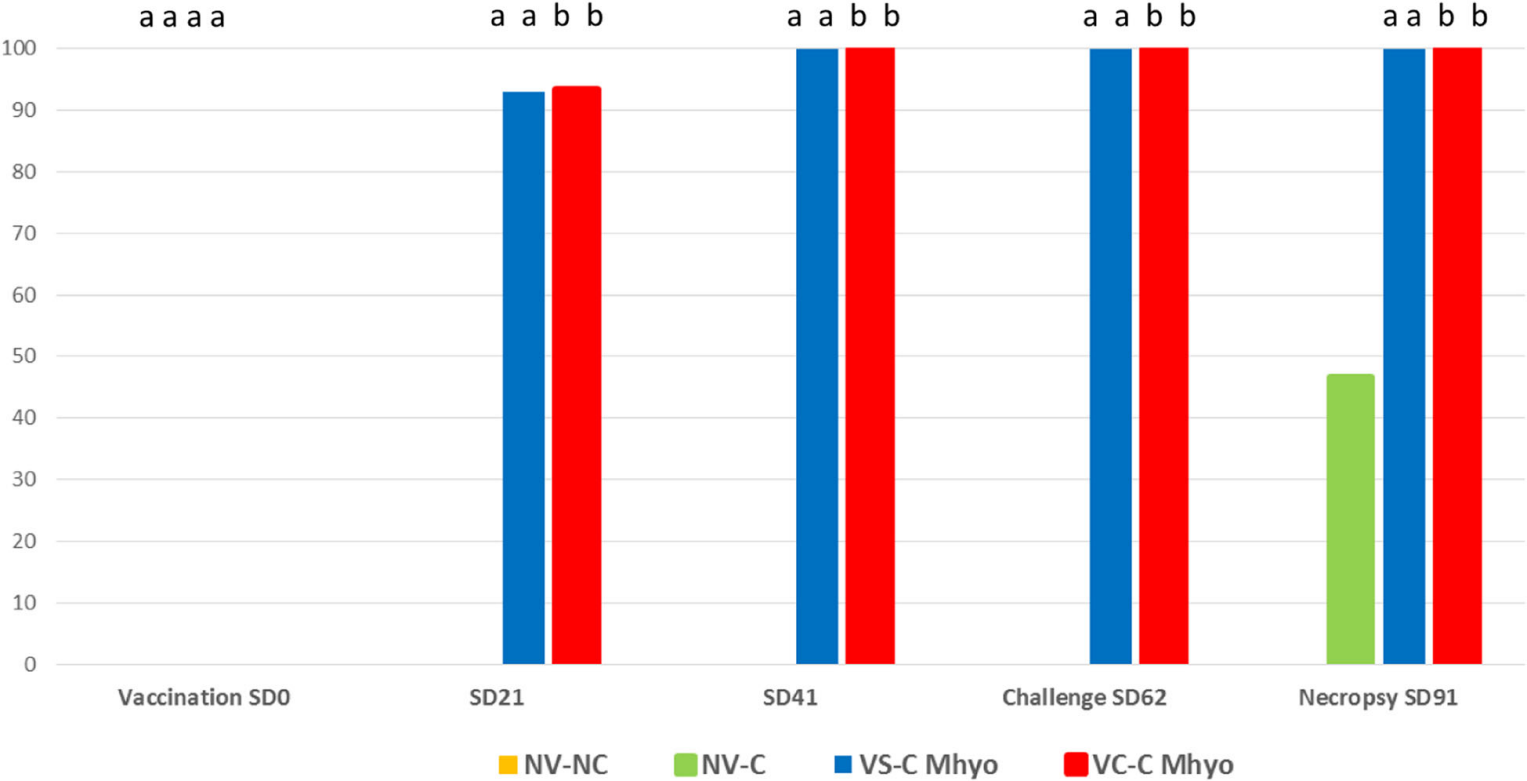
Percentage of *M. hyopneumoniae* seropositive animals per treatment and sampling point. Different superscript letters mean *p* < 0.05

Percentages of Mhyo seropositive animals are represented in Fig. 4. All animals included in the study were seronegative by ELISA prior to the start of the study. Pigs from the NV-NC group remained seronegative through the study. In the NV-C Mhyo group, seroconversion was observed on necropsy day in which 7 out of the 15 animals were seropositive. In both vaccinated groups, almost all animals were seropositive from 3 weeks post vaccination (SD21) onwards. Statistically significant differences between both vaccinated versus both non-vaccinated groups were observed on SD21, SD41, SD62 and SD91 (necropsy day).

#### Mhyo macroscopic and microscopic compatible lung lesions assessment

None of the animals included in the NV-NC group showed macroscopic Mhyo compatible lung lesions (cranio-ventral pulmonary consolidation, CVPC). From the 43 endotraqueally Mhyo inoculated animals (two animals died before necropsy), 25 (58%) animals showed CVPC (mean: 3.36; min 0.12-max 38.63). Animals from the NV-C Mhyo group showed a significantly higher CVPC score (Table 3) than the animals from both vaccinated groups.

**Table 3:** Total number (percentage) and mean (minimum and maximum) macroscopic score of animals showing CVPC or CVPC+BIP per treatment group

Treatment	Animals showing CVPC		Animals showing CVPC + BIP	
	Proportion of animals with lesions (%)	Mean CVPC Score (Min-Max)	Proportion of animals with lesions (%)	Mean CVPC Score (Min-Max)
**NV-NC**	0/3 (0)	0 ^a^	0/3^a^ (0)	0 ^A^
**NV-C Mhyo**	12/15 (80)	6.23^b^ (0.12–38.63)	10/15^b^ (66.6)	7.26^B^ (38.63–0.60)
**VS-C Mhyo**	7/14 (50)	0.62^a^ (0.18–1.20)	2/14^a^ (14.3)	0.75^A^ (1.20–0.30)
**VC-C Mhyo**	6/14 (43)	0.84^a^ (0.12–2.48)	1/14^a^ (7.2)	0.53^A^

Different low case letters in superscript within a column mean *p* < 0.05; different capital letters in superscript within a column mean *p* < 0.1

From these 25 animals showing CVPC, 12 did not show broncho-interstitial pneumonia (BIP) (10 received a microscopic score 0 and the remaining ones were scored 1). Therefore, macroscopic lesions observed in these 12 animals were re-scored 0 for the gross evaluation purposes. In consequence the number of animals showing CVPC due to BIP was 13 out of 43 (30%). From these 13, the number of animals showing CVPC was significantly higher in the NV-C (*n* = 10, 77%) group than the ones from VS-C Mhyo (*n* = 2, 14%) and VC-C Mhyo (*n* = 1, 7%) groups. When considered CVPC+BIP, animals from the NV-C Mhyo group showed a higher CVPC score (Table 3) than the animals from both vaccinated groups (*p* < 0.1).

**Fig. 5:**
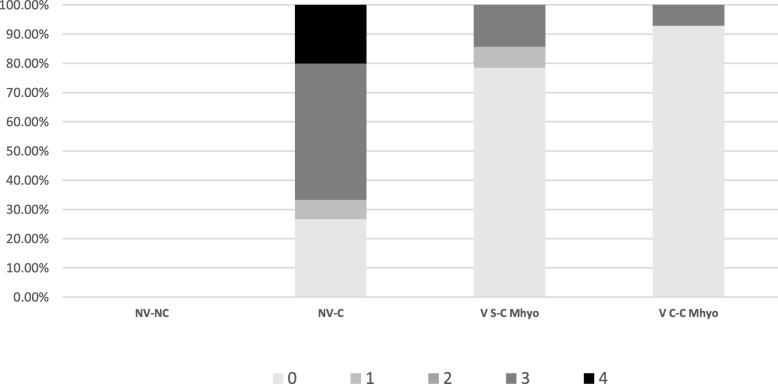
Percentage of animals showing different microscopic lung lesion score (0–4) per treatment group. NV-C had significantly higher number of animals showing a microscopic score ≥ 3 than both vaccinated groups

From the 13 animals showing BIP + CVPC, 10 were microscopically scored as 3 (7 from NV-C Mhyo; 2 from VS-C Mhyo and 1 from VC-C Mhyo) and 3 were scored as 4 (all of them from NV-C Mhyo group) (Fig. 5). The NV-C Mhyo group had a significantly higher number of animals scoring ≥3 than the vaccinated groups. No significant differences on the number of animals scored ≥3 between vaccinated groups were found.

## Discussion

PCV-2 and Mhyo combined vaccination strategies (RTM and RTU) have interested both veterinarians and farmers for several years, since they decrease the cost of labour. Besides the lower labour expenses, other factors such as animal welfare (lesser number of injections and manipulations) and lower risk of other pathogen transmission (through syringes and needles) account importantly as benefits of a single injection of two vaccine products. In some cases, however, a suspicion of lesser effectivity of these combined products has been suggested [15], posing doubts on their overall benefit. In fact, these later authors attributed such questionable efficacy to the variation of the epidemiology of respiratory diseases among farms and regions [15], claiming about the scarcity of comparative data on single-shot two valence vaccines.

In the present study, the efficacy of Hyogen® and Circovac® when applied separately or combined (RTM) was assessed by means of Mhyo or PCV-2 experimental challenges.

The outcome of the present PCV-2 experimental infection was subclinical as no PCV-2 clinical signs were observed. These results are in line with previous PCV-2 experimental inoculations [16, 17]. In this scenario, both PCV-2 vaccine application strategies showedhigher PCV-2 ELISA S/*P* values after vaccination than the NV-C group. Although statistically significant differences on PCV-2 ELISA S/P values between both vaccinated groups were seen on SD70 and SD76, both groups reached the maximum and similar serologic values on the necropsy day. The reason behind the fact that the overall values at most of samplings were higher in the VS-C-PCV-2 group compared to the VC-C-PCV-2 is unknown. Despite it cannot be ruled out that combination of both products may exert certain deleterious effect on PCV-2 seroconversion, the virological outcome was not significantly different among these groups. Additionally, both vaccinated groups (VS-C PCV-2 and VC-C PCV-2 groups) were able to reduce (being statistically significant on necropsy day) the percentage of PCV-2 QPCR positive serum samples when compared to the one of the NV-C group. Indeed, vaccinated animals (either separately or RTM) had a numerically lower AUC than non-vaccinated ones. This reduction of PCV-2 infected pigs in both PCV-2 vaccinated groups was accompanied by a lower number of animals harbouring PCV-2 in tissues when compared to their non-vaccinated counterparts. Moreover, results obtained in animals vaccinated with the PCV-2 separate application resembled the ones obtained using the same vaccine in 3-week-old animals under experimental PCV-2 subclinical infections [18, 19].

On the other hand, vaccination against Mhyo, either separately or in a RTM combination with the PCV-2 vaccine, resulted in a significantly higher percentage of seropositive animals 3 weeks after vaccination, a significant reduction of the number of animals showing *M. hyopneumoniae* macro and microscopic lesions as well as their extension (macroscopic score) and severity (microscopic score) when compared to non-vaccinated animals. Seroconversion results and the macroscopic lung lesion reduction are in agreement when compared with experimental inoculations in which the same Mhyo vaccine employed in this study was used solely [20].

Taken all together, obtained results point out that the two vaccines used in this study (either by separate, which represents the current licensing use, or in combination, off-label scenario), offer similar results from virological, immunological and pathological points of view under experimental conditions. Therefore, these data point out the possibility of combining the abovementioned vaccine products in one single injection in these farms where both vaccines are applied in a similar age.

## Conclusion

The present study expands the knowledge on the possibility of using two separate Mhyo and PCV-2 commercial vaccines as a RTM product, which offered equivalent virological/immunological and pathological outcomes as compared to these vaccines by separate. Next step should be directed towards the demonstration of such efficacy under field conditions.

## Materials and methods

### Animals and housing

At approximately 1 week of age (WOA), blood samples from 129 male non-castrated piglets born from PCV-2 non-vaccinated sows were obtained from a Mhyo negative farm. These blood samples were analysed for the presence of PCV-2 by QPCR assay to rule out any positive piglets. Those QPCR PCV-2 negative samples were tested for antibodies against Mhyo and PCV-2 using commercial ELISAs. Then, 96 two-week old male piglets that resulted negative to PCV-2 QPCR, with low levels of PCV-2 antibodies (Sample/Positive (S/P) ratio ≤ 0.8 in Ingezim Circo IgG ELISA) and seronegative against Mhyo (percentage of inhibition > 50%) were selected and transported to an IRTA experimental farm.

### Vaccine and placebo products

The vaccine tested products were a commercial inactivated PCV-2a based vaccine (CIRCOVAC®, CEVA, France; batch number L438978 and expiration date: 03/05/2018) with an oil/water adjuvant and a commercial inactivated Mhyo vaccine (Hyogen®, CEVA, France; batch number 0904FT1B and expiration date: 11/07/2018) with oil adjuvant. Phosphate-buffered saline (PBS) (Lonza, Spain; batch number 7 MB052 and expiration date: 03/2019) was used as placebo.

### Inoculum

PCV-2 strain Sp-10-7-54-13 (PCV-2b genotype) was used as PCV-2 inoculum [21]. This strain was isolated from the lymphoid tissues of a field case of PCVDs in 2006 in Spain. The inoculum preparation and the post-challenge titration were as described previously [21].

A fresh culture of Mhyo field strain 281 was used as Mhyo inoculum. This strain was isolated in 2006 from a lung of a slaughtered Spanish pig showing CVPC. The inoculum preparation and the post-challenge titration were as described previously [22]. Absence of *M. hyorhinis* and *M. hyosynoviae* was confirmed by specific PCR.

### Experimental design and samplings

Upon arrival at the experimental facilities (SD-5), 96 animals were distributed in different groups (Table 4) according to the body weight. The 45 pigs with the lowest S/P PCV-2 values at 1 WOA were distributed in the 3 groups of the PCV-2 study. Similarly, 45 animals were randomly included in the Mhyo study and distributed in 3 groups. The 6 remaining pigs were included in the NV-NC group. Animals of different treatment groups were allocated in different pens and rooms.

**Table 4:** Experimental design of the study

Treatment	N	Vaccination		Challenge	Necropsy PCV-2	Necropsy Mhyo
		Left Side of the Neck (volume and route)	Right Side of the Neck (volume and route)			
**NV-NC**	6	PBS (2.5 mL IM)	–	PBS	PCV-2 lesion assessment	Mhyo lung lesion scoring
**NV-C/PCV-2**	15	PBS (2.5 mL IM)	–	PCV-2b	PCV-2 lesion assessment	–
**VS-C/PCV-2**	15	Circovac (0.5 mL IM)	Hyogen (2.0 mL IM)	PCV-2b		–
**VC-C/PCV-2**	15	Circovac + Hyogen (2.5 mL IM RTM)	–	PCV-2b		–
**NV-C/Mhyo**	15	PBS (2.5 mL IM)	–	Mhyo	–	Mhyo Lung lesion scoring
**VS-C/Mhyo**	15	Circovac (0.5 mL IM)	Hyogen (2.0 mL IM)	Mhyo	–	
**VC-C/Mhyo**	15	Circovac + Hyogen (2.5 mL IM RTM)	–	Mhyo	–	

*NV-NC* Non-vaccinated Non-challenged, *NV-C* Non-vaccinated-Challenged animals, *VS-C Challenged (with PCV2 or Mhyo)* Separate vaccination, *VC-C (with PCV2 or Mhyo)* Combined (RTM) vaccination

At approximately 21 ± 3 days of age (3 WOA), animals from non-vaccinated (NV) groups (NV-NC [non-challenged], NV-C PCV-2 [challenged with PCV-2] and NV-C Mhyo [challenged with Mhyo]) were injected intramuscularly with 2.5 mL of phosphate-buffered saline (PBS) at the left side of the neck. Animals from group VS (separated vaccination)-C PCV-2 and VS-C Mhyo were intramuscularly vaccinated, following manufacturer’s instructions with 0.5 mL of Circovac® and with 2 mL of Hyogen® at the left and right side of the neck, respectively. Animals from group VC (combined vaccination)-C PCV-2 and VC-C Mhyo were intramuscularly vaccinated with 2.5 mL of a RTM combined vaccine (Circovac® + Hyogen®) at the left side of the neck. The RTM vaccine was prepared as follows: first Circovac® was re-constituted as per manufacturer’s instructions and then 10 mL of Circovac® were mixed with 40 mL of Hyogen® in a sterile container.

At SD62 (approximately 12 WOA) animals were weighted, sampled and challenged as follows: NV-NC pigs were challenged with PBS (3 mL intranasally in three of them and 5 mL endotracheally in the other three); animals from C PCV-2 groups were intranasally challenged with 3 mL of inoculum 10^5.4^ TCID50/mL of PCV-2b strain Sp-10-7-54-13; and, finally, animals from C Mhyo groups were endotracheally challenged with 5 mL per day of 10^7.5^ (first day) and 10^8^ (second day) PCR_50_/mL fresh culture of a Mhyo isolate on two consecutive days. PCV-2 intranasal inoculation was performed as described elsewhere [21]. Mhyo endotracheal inoculation was done as reported previously [22].

After inoculation, pigs were clinically examined on a daily basis focused on respiratory signs such as dyspnoea and coughing. Observations consisted of pen-side visual assessment of animals by experienced animal care-takers.

At SD83 (21 days post PCV-2 challenge), the 3 piglets mock-challenged by intranasal route from group NV-NC and all pigs from PCV-2 groups were humanely euthanized with an overdose of sodium pentobarbital. At necropsy, animals were firstly bled and weighted and then different tissue samples (tonsils, tracheobronchial lymph nodes, mesenteric lymph nodes and superficial inguinal lymph nodes) were collected. At SD91 (28 days post-Mhyo challenge) the 3 remaining piglets from group NV-NC and all pigs from Mhyo groups were bled, weighted and euthanized as explained before. At necropsy and subsequent pathological analyses, lungs were scored in a blinded fashion for gross and microscopic lesions related to Mhyo infection.

The study procedure was referenced 9998 by Animal Experimentation Ethics Committee of the Generalitat de Catalunya (*Departament de Territori i Sostenibilitat, Direcció General de Polítiques Ambientals i Medi Natural*).

### Samples

Blood samples were collected individually from all piglets, at the source farm, prior to the start of the study for screening and inclusion purposes (SD-14), at SD0 (vaccination day) and then every 3 weeks post-vaccination (SD21 and SD41). For PCV-2 challenged animals, samples were taken on the day before challenge (SD62), SD70, SD76 and SD83 (necropsy). For Mhyo challenged animals, samples were collected on the day before challenge SD62 and at necropsy on SD91.

Samples were transferred to the laboratory site at 4–8 °C. Blood samples were centrifuged and serum was obtained. Until use, sera were stored at − 20°C. The period between blood sampling and storage did not exceed 24 h.

### Post-mortem examination

#### Mhyo lung lesion scoring

At necropsy, photographs were taken from both sides of the lung. Afterwards, extension of Mhyo-compatible lung lesions (CVPC) were assessed according to the score recommended for the European Pharmacopoeia (Ph. Eur. monograph no.2448). Briefly, for each lung, each individual lobe was assessed as percentage of lung with lesions resulting from the Mhyo challenge. Afterwards, the proportion of affected lung area was multiplied by the relative weight of each lobe [22]. In addition, lung samples from pneumonic areas were collected and fixed by immersion in 10% buffered formalin for histopathological examination. Fixed tissue samples were dehydrated and embedded in paraffin blocks, stained with haematoxylin-eosin stain, and examined for lesions suggestive of Mhyo infection. Mhyo lung lesion scoring was performed in a blinded fashion taking into account two pathological criteria: (1) presence of gross CVPC and (2) confirmation of such gross lesions as BIP, as previously described [16]. Absence of lesion was scored 0; lesions characterized by inflammation non-suggestive of enzootic pneumonia were scored 1 and 2; scores 3 and 4 were considered suggestive of Mhyo infection. Score 3 consisted of lesions with perivascular and peribronchiolar lymphoplasmacytic hyperplasia, pneumocyte type II hyperplasia and oedema fluid in the alveolar spaces with neutrophils, macrophages and plasma cells. Score 4 was considered when lesions with the characteristics of score 3 together with the presence of evident peribronchial and perivascular lymphoid follicles were observed. Scores 3 and 4 were considered highly compatible with lesions of Mhyo infection. Therefore, overall Mhyo-like lesions were evaluated for the existence of EP-like gross lesions and the score was kept only if histopathological evaluation confirmed the presence of BIP. Those cases showing EP-like gross lesions, but with microscopic evidence only of pulmonary collapse (atelectasis) were scored as 0 on the gross evaluation.

#### Histopathologic studies and PCV-2 immunohistochemistry

Tonsil and tracheo-bronchial, mesenteric and inguinal superficial lymph nodes were fixed by immersion in 10% buffered formalin, dehydrated and embedded in paraffin blocks (one block per pig with all four tissues). From each paraffin block, two consecutive 4 μm thick sections were cut. One section was stained with the haematoxylin-eosin (HE) stain and examined for lesions compatible with PCV-2 infection, including lymphocyte depletion (LD) and histiocytic infiltration (HI). The other section was processed for immunohistochemistry (IHC) for PCV-2 antigen detection. Lymphocyte depletion, histiocytic infiltration and the amount of PCV-2 antigen were scored from 0 (no lesions/no staining) to 3 (severe lesions/widespread antigen distribution). The score for each lymphoid tissue sample was calculated as follows: score for lymphocyte depletion plus score for histiocytic infiltration plus score for IHC. Afterwards, a global histopathological score per pig was calculated by summing the individual scores of all four lymphoid tissue samples [23].

### Detection of antibodies against PCV-2

Presence of antibodies against PCV-2 in blood were tested with the commercial ELISA Ingezim Circo IgG 11. PCV.K1 kit (Ingenasa, Madrid, Spain). Results were expressed as mean S/*P* values.

### Detection of antibodies against Mhyo

Presence of antibodies against Mhyo in serum samples were tested with the commercial competitive ELISA IDEIA *M.hyopneumoniae*, EIA kit (ThermoFisher Scientific. Hampshire, UK). Results were expressed as percentage of positive samples (percentage of inhibition [PI] < 50%).

### Detection and quantification of PCV-2 DNA

DNA was extracted from 200 μL of serum by using the MagMAX™ Pathogen RNA/DNA Kit (Thermo Fischer Scientific Baltics. Vilnius, Lithuania) following the manufacturer’s instructions. The DNA obtained was suspended in 90 μL of elution solution. The commercial QPCR kit VetMAX™ Porcine PCV2 Quant Kit (Applied Biosystems, Lisseu, France) was used to detect and quantify PCV-2 DNA in serum samples. PCV-2 QPCR were expressed as percentage of QPCR positive samples (including also the samples QPCR positive but below limit of quantification) and mean PCV-2 copies/mL of serum (including only the samples within the range of quantification). The AUC of the PCV-2 load during the post-challenge period (from SD70 to SD86) per treatment group was calculated following the trapezoidal rule [24].

### Statistical analyses

To compare baseline characteristics between groups (PCV-2 S/P ELISA values at 1 WOA and body weight at arrival day [SD-5]) ANOVA was performed.

Two independent statistical analyses (one per each challenge experiment) were performed. In each analysis, comparison between the NV-NC, NV-C and both V (separated vs combined) groups were done. These comparisons were conducted considering all 6 NV-NC animals for all samplings but SD91 (necropsy day for Mhyo) in which only 3 animals remained (the other three were necropsied on SD84, when PCV-2 challenged pigs were euthanized).

Average daily weight gain between SD-5 to SD62, SD-5 to SD91 and SD62 to SD91 was calculated for each treatment group. The analysis of quantitative variables (PCV-2 ELISA values, body weight, ADWG, QPCR, AUC and macroscopic lung score) was performed using an ANOVA or Kruskal-Wallis test. For categorical variables (percentage of positivity by Mhyo ELISA, percentage of positivity by PCV-2 QPCR, microscopic Mhyo score and PCV-2 IHC and CVPC presence), the Chi square test or likelihood ratio test were used. For the analysis of total PCV-2 IHC score, a logistic regression model was used. Pairwise comparisons were corrected for multiplicity of contrasts.

Additionally, each response variable was analysed using linear mixed models. Sow was included as a random effect, and group and variables that had lack of homogeneity between groups as fixed effects. A normal distribution was considered for the analysis of ELISA and body weight, a Binary distribution for the analysis of microscopic scores and a Poisson distribution for the analysis of macroscopic scores.

All analyses were performed with software SAS v9.4 (SAS Institute Inc., Cary, NC, USA). The significance level was set at *p* < 0.05.

## Data Availability

The datasets used and analyzed during the trial are available from the corresponding author on request.

## Abbreviations

Mhyo: *Mycoplasma hyopneumoniae*
PCV-2: *Porcine circovirus 2*
RTM: Ready-to-mix
RTU: Ready-to-use
WOA: Weeks of age
NV: Non-vaccinated
V: Vaccinated
NC: Non-challenged
VC: Combined vaccination
VS: Separate vaccination
ADWG: Average daily weight gain
QPCR: Quantitative Polymerase Chain Reaction
ELISA: Enzyme-linked immunosorbent assay
S/P: Sample/Positive
PI: Percentage of Inhibition
PBS: Phosphate-buffered saline
CVPC: Cranio-ventral pulmonary consolidation
BIP: Broncho-interstitial pneumonia
HE: Haematoxylin-eosin
LD: Lymphocyte depletion
HI: Histiocytic infiltration
IHC: Immunohistochemistry
